# Antibacterial Composites Based on Alginate/Egg White and ZnO Nanoparticles with the Addition of Essential Oils

**DOI:** 10.3390/gels11060459

**Published:** 2025-06-16

**Authors:** Adrian-Ionuț Nicoară, Adelina Valentina Anton, Roxana Doina Trușcă, Alexandra Cătălina Bîrcă, Cornelia-Ioana Ilie, Lia-Mara Dițu

**Affiliations:** 1 Department of Science and Engineering of Oxide Materials and Nanomaterials, Faculty of Chemical Engineering and Biotechnologies, National University of Science and Technology Politehnica Bucharest, 1-7 Gh. Polizu, 011061 Bucharest, Romania; adrian.nicoara@upb.ro; 2 National Research Center for Micro and Nanomaterials, National University of Science and Technology Politehnica Bucharest, 313 Spl. Independenţei, 060042 Bucharest, Romania; roxana_doina.trusca@upb.ro (R.D.T.); alexandra.birca@upb.ro (A.C.B.); 3 Faculty of Medical Engineering, National University of Science and Technology Politehnica Bucharest, 313 Spl. Independenţei, 060042 Bucharest, Romania; antonadelina07@gmail.com; 4 Department of Botany and Microbiology, Faculty of Biology, University of Bucharest, 1-3 Portocalilor, 060101 Bucharest, Romania; lia-mara.ditu@bio.unibuc.ro; 5 MICROGEN Research Centre, Faculty of Biology, University of Bucharest, 1-3 Portocalilor, 060101 Bucharest, Romania

**Keywords:** egg white, alginate composite, wound dressing, antibacterial materials

## Abstract

A series of hydrogels containing sodium alginate at different concentrations (2%, 3%, and 4%) and egg white were prepared through ionic cross-linking with calcium chloride (CaCl_2_) to obtain composite dressing materials. ZnO nanoparticles coated with eucalyptus or lavender essential oil were introduced into the hydrogel matrix to enhance antibacterial properties. The resulting hydrogels were freeze-dried to enhance mechanical properties, increase the porosity of the dressing, and facilitate further evaluations. A variety of analytical methods, including scanning electron microscopy (SEM), X-ray dispersive spectroscopy (EDS), and Fourier transform infrared spectroscopy (FT-IR) were employed to characterize the composites. The developed composites exhibited high porosity and a swelling degree exceeding 200% after 3 days. Additionally, water absorption capacity increased with higher alginate concentrations in the samples. Furthermore, they demonstrated significant antibiofilm activity against *Staphylococcus aureus*, *Enterococcus faecalis*, and *Escherichia coli*, with the samples containing 4% alginate showing the best results.

## 1. Introduction

Skin and tissue infections are well-defined as damage produced by the pathogens, with variable resistance and severity [1,2]. The most characteristic aspects of wound infections are their ability to form a biofilm, which affects the healing processes by reducing the effectiveness of antibiotics and promoting resistance of the microorganisms to treatment [3]. Moreover, the wound-healing processes are disrupted and delayed [4]. The commonly isolated bacterial species from chronic wounds include *S. aureus*, *P. aeruginosa*, *E. coli*, *Enterococcus* spp., *Corynebacterium* spp., *Streptococcus* spp., *Klebsiella* spp., and *Acinetobacter baumannii* [3,5].

Nanotechnology enhances wound dressings by utilizing materials loaded with targeted drug delivery systems to improve the healing process [6]. One therapeutic agent can be ZnO, defined by the Food and Drug Administration as a substance Generally Recognized as Safe [7]. Recent studies have highlighted the antimicrobial properties of ZnO nanoparticles (NPs) [8,9,10,11], positioning them as a promising option for various applications [12,13,14,15,16,17,18,19]. The antimicrobial activity of ZnO NPs can be explained by the effect of Zn^2+^ ions, which trigger the production of reactive oxygen species, respectively, damaging several structures of bacterial cells [20]. Additionally, the sensitivity of microorganisms to the action of ZnO NPs is influenced by the small size and shape of the nanoparticles [21]. Alginate and albumin are among the most commonly used materials in wound dressings because of their biocompatibility and antimicrobial properties [22,23].

Alginate is an anionic polysaccharide found in nature, the primary source of extraction being the cell wall of brown algae. Sodium alginate (NaC_6_H_7_O_6_) represents alginic acid’s sodium salt and contains approximately 30–60% alginic acid.

Its property of swelling in contact with moist environments makes it possible to use it in controlled drug-release systems. The absorption capacity is obtained by forming a strong hydrophilic gel, which limits wound exudates and minimizes bacterial contamination. Once alginate dressings are applied to the wound, the ions present in the alginate are exchanged with the blood to form a protective film. A key characteristic of alginate hydrogels is their non-adhesive nature to wound tissues, allowing for pain-free removal without creating small lesions on the wound surface [24].

Compared to other natural biomaterials, albumin exhibits a high bioactive character, is easy to manipulate, and has lower production costs than other proteins like collagen or fibronectin [25]. It has also been demonstrated that albumin can serve as an interface between the scaffold and cells, enhancing the interaction between the two components by acting as a mediator, with the efficiency of attaching cellular material being even higher than that of collagen or fibronectin [26]. Regarding the biomedical applications of egg white, it has been known for centuries for its use in the treatment of various conditions, such as wound treatment; in the case of burns, it has antibacterial, anti-inflammatory effects, and stimulates cell proliferation [25]. Due to its easy processing, egg white can generate a wide range of biomaterials whose mechanical properties and biological response from the seeded molecules can be controlled using biotechnology. In fact, egg white proteins exhibit versatile functional technical properties, such as foaming ability, emulsifying property, and gel formation upon heating. As a result of these processes, they can be shaped into porous scaffolds, hydrogels, films, fibers, and particles [27]. Research on using egg white ointment to treat second-degree burns was conducted by Simin Jahani et al. [28] in 2019.

Essential oils have a wide range of applications, with the most popular being in aromatherapy. In the medical industry, essential oils have multiple applications due to their bactericidal, virucidal, and fungicidal properties [29]. Eucalyptus essential oil is isolated and extracted from the dried leaves of *Eucalyptus citriodora* or other eucalyptus species. It possesses various therapeutic properties, including analgesic, antibacterial, antioxidant, and anti-inflammatory activities reported in the literature. Its antibacterial activity has been shown to be due to a chemical component called cineole. [30]. Lavender essential oil is extracted from the flowering tops of lavender (*Lavandula angustifolia Mill*) and is used in traditional medicine for its sedative, antidepressant, antimicrobial, antifungal, and analgesic properties [31]. In dermatology, lavender essential oil has been used to treat wounds, eczema, and psoriasis [32]. Hiroko-Miyuki Mori et al. [33] analyzed the potential of applying lavender essential oil in wound healing, demonstrating that the surface area of wounds treated with this oil decreased significantly.

This study aims to design a freeze-dried hydrogel dressing for the treatment of burns composed of sodium alginate, egg white, ZnO NPs, and eucalyptus or lavender essential oils to prevent external wound infection pathogens. These materials were selected because of their properties possess, which have been previously studied in scientific articles. Egg white is a rich source of albumin and is well known for its applications in controlled drug release [34]. Firstly, ZnO nanoparticles coated with eucalyptus or lavender essential oil were synthesized and characterized, followed by the dressing development. The study’s novelty consists of using albumin in combination with sodium alginate to improve the composite’s bioactivity and cellular adhesion. Moreover, the anti-adherent/antibiofilm capacity of alginate/egg white-ZnO membranes was assessed against the most frequently isolated strains from wounds (two drug-resistant Gram-positive and one standard Gram-negative bacteria). Furthermore, the viability of human umbilical vein endothelial cells under the action of composite materials was evaluated.

## 2. Results and Discussion

### 2.1. ZnO Nanoparticles

**Figure 1:**
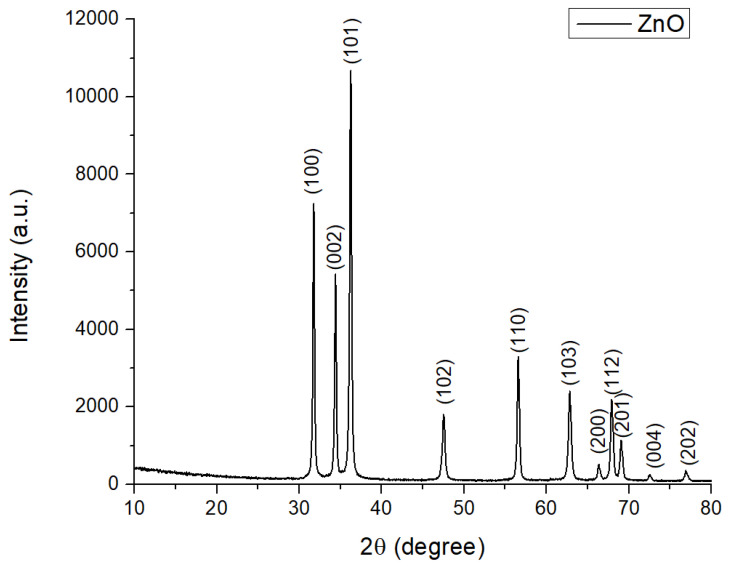
XRD patterns on ZnO samples obtained by the combustion method.

The XRD analysis was performed in order to characterize the synthesized materials in terms of their crystallinity as well as their component phases and was carried out over the range of angles 2θ = 10–80° (Figure 1). The crystalline phase, identified using the ICDD 04-007-1614 file, is hexagonal ZnO (P63mc space group) crystallized predominantly along the direction given by the Miller indices corresponding to the 101 plane. The high intensity and the shape of the diffraction peaks indicate a high crystallinity degree of this phase, while the average crystallite size, determined by the Scherrer equation, is approximately 47 nm. No diffraction maxima corresponding to other elements (impurities) were observed, which confirms that the obtained powder is a pure phase [35].

**Figure 2:**
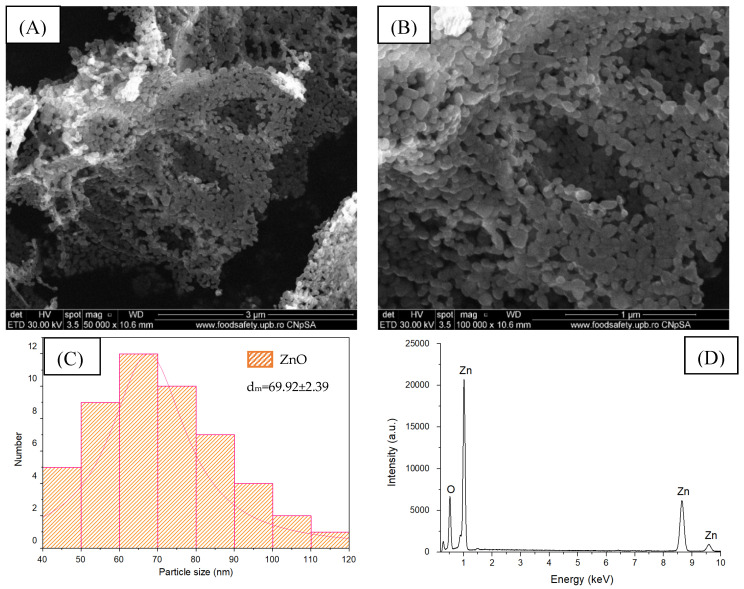
SEM image of ZnO nanoparticles at 50,000× (**A**) and 100,000× (**B**) magnification, size distribution (**C**), and EDS (**D**).

Scanning electron microscopy was performed to highlight the synthesized sample morphology. The micrographs were obtained at different magnifications, 50,000× and 100,000×, and the results obtained are presented in Figure 2A,B.

From the SEM images of ZnO NPs, it can be observed that the particles show a quasi-spherical and irregular morphology with a size in the range of 40–120 nm and a high tendency to agglomerate due to their size and specific surface. The histogram in Figure 2C represents the particle size distribution in the ZnO powder and was realized by measuring more than 50 nanoparticles, resulting in a mean particle size equal to 69.92 ± 2.39 nm with a unimodal size distribution. The energy dispersive spectra (EDS) obtained on the ZnO samples obtained by the combustion method (Figure 2D) indicate the presence of two characteristic atoms: ZnO (54.24 wt.%) and O (45.76 wt.%). The absence of energy maxima characteristic of other chemical elements indicates these samples’ high degree of purity.

### 2.2. Alginate/Egg White–ZnO Composite

**Figure 3:**
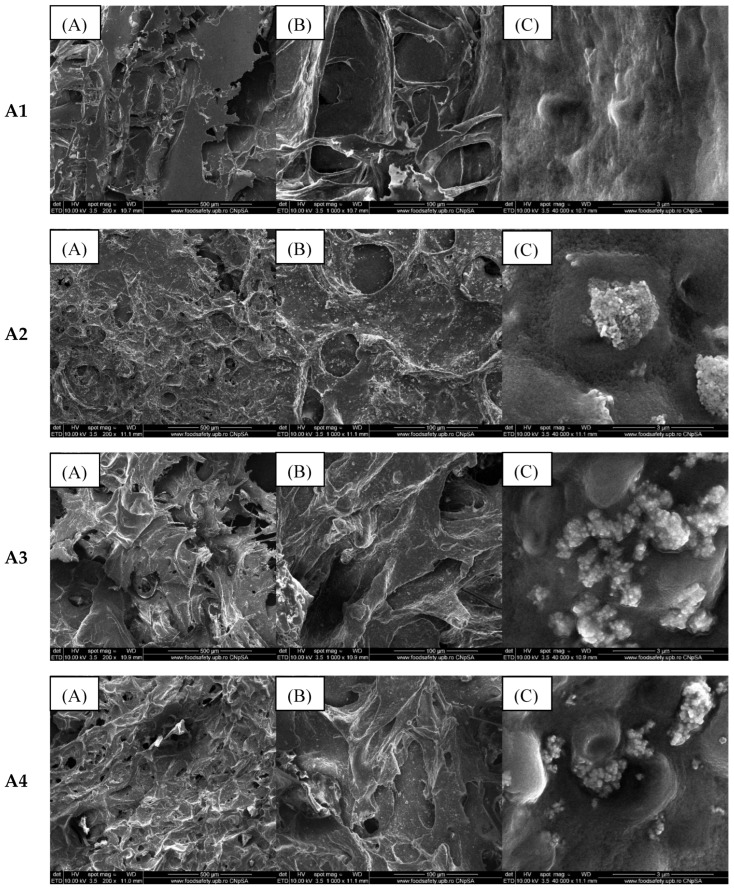
SEM images of composite samples containing 2% alginate and egg white (**A1**), 2% alginate, egg white and ZnO (**A2**), 2% alginate, egg white, and ZnO coated with lavender oil (**A3**) and eucalyptus oil (**A4**) at various magnifications ((**A**) 200×, (**B**) 1000×, and (**C**) 40,000×).

The composite materials obtained by freeze-drying the homogeneous mixture of alginate, egg white, and zinc NPs (with or without adding essential oils) were characterized morpho-structurally and compositionally using scanning electron microscopy. Figure 3 presents the morphology investigated using secondary electrons for the samples noted in Table 2 with the letter A.

**Figure 4:**
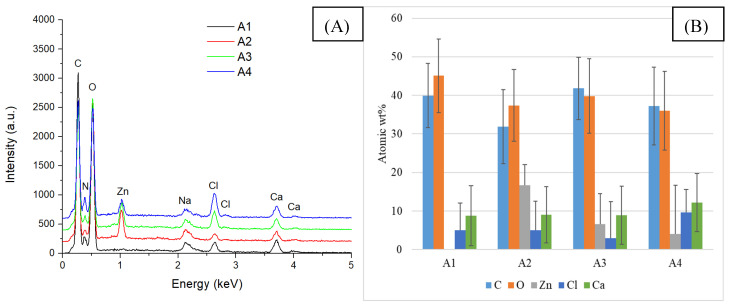
EDS analysis (**A**) and chemical composition (**B**) of composite samples included in group A.

According to the SEM images, all three samples show a porous structure with interconnected porosity due to the hydrogels’ freeze-drying process. The addition of ZnO NPs causes an increase in the porosity of the samples, most likely due to their distribution in the polymer matrix. As shown in Figure 3, the absence of essential oil on the ZnO nanoparticles’ surface induces their accumulation in certain sample areas. This is also highlighted by the EDS analysis presented in Figure 4.

The chemical elements identified in all samples are those specific to sodium alginate and egg white (C, O, Na) as well as zinc oxide NPs (Zn, O). The presence of calcium and chlorine is due to the CaCl_2_ solution that was used for crosslinking. The highest weight percentage is C and O because these elements are present in the chemical structure of both alginate and egg white proteins.

**Figure 5:**
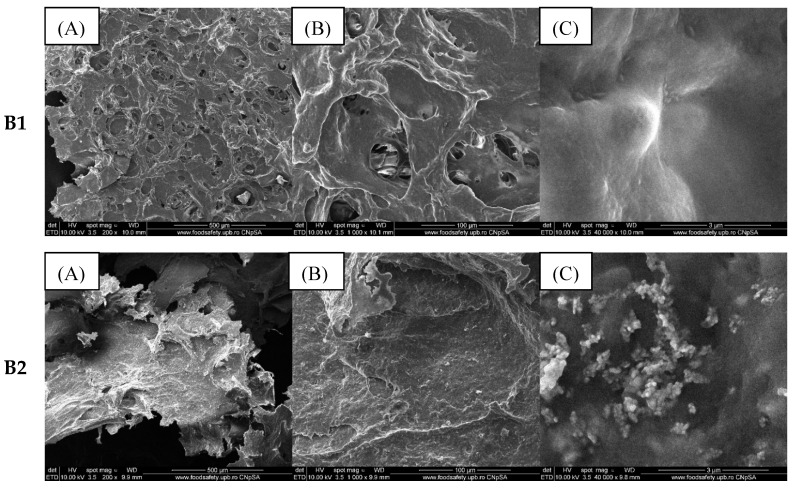

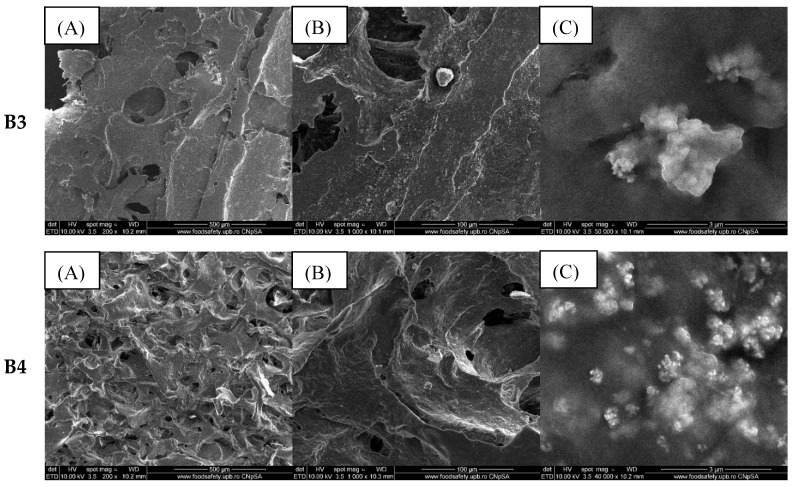
SEM images of composite samples containing 3% alginate and egg white (**B1**), 3% alginate, egg white and ZnO (**B2**), 3% alginate, egg white, and ZnO coated with lavender oil (**B3**) and eucalyptus oil (**B4**) at various magnifications ((**A**) 200×, (**B**) 1000×, and (**C**) 40,000×).

Topographic analysis of samples containing 3% alginate, egg white, and coated/uncoated ZnO NPs (group B) was presented in Figure 5.

It is observed that ZnO NPs were embedded throughout the entire mass of the composite, which was relatively uniformly distributed as agglomerates due to the small size of the nanoparticles.

**Figure 6:**
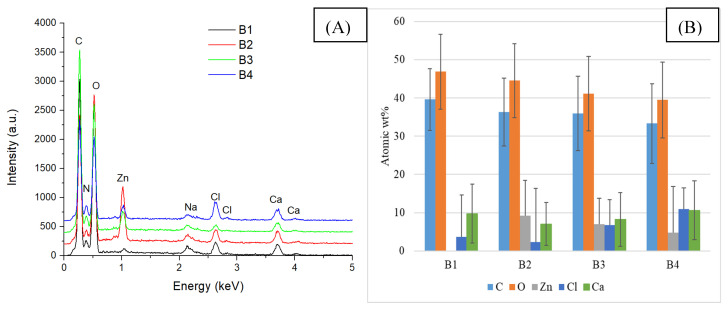
EDS analysis (**A**) and chemical composition (**B**) of composite samples included in group B.

Increasing the alginate concentration to 3% (group B) does not affect the distribution of ZnO NPs in the composite mass. The EDS spectra and element distribution, shown in Figure 6, are similar to those in group A (with 2% alginate).

**Figure 7:**
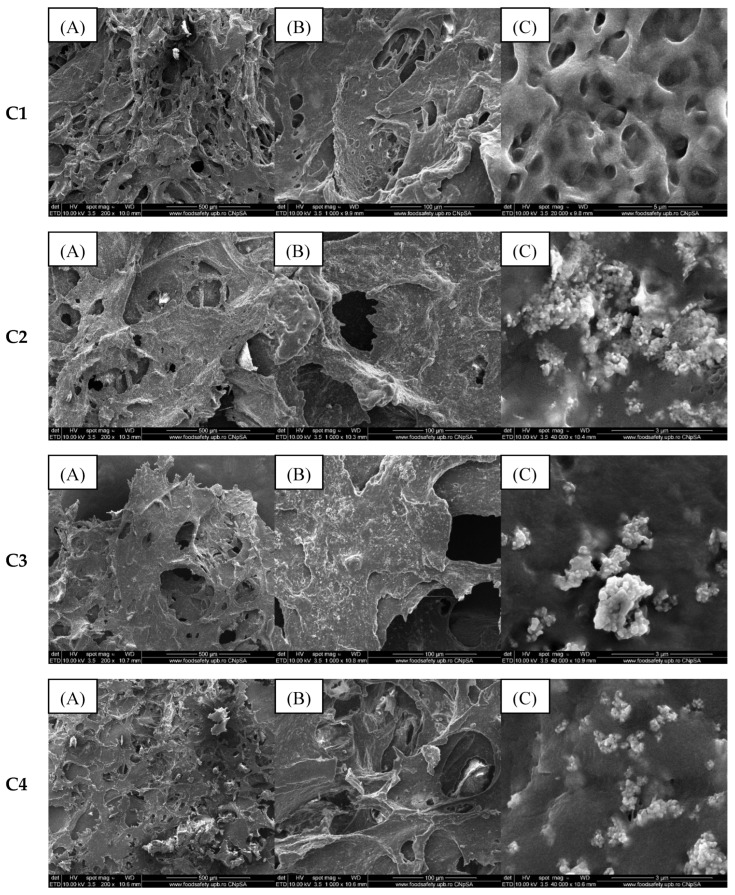
SEM images of composite samples containing 4% alginate and egg white (**C1**), 4% alginate, egg white and ZnO (**C2**), 4% alginate, egg white, and ZnO coated with lavender oil (**C3**) and eucalyptus oil (**C4**) at various magnifications ((**A**) 200×, (**B**) 1000×, and (**C**) 40,000×).

In contrast, as the alginate concentration increases (up to 4%), as is the case for samples in group C, the scanning electron microscopy images (Figure 7) show an increase in matrix roughness determined by the freeze-drying process.

In these SEM images, compared to the previous ones in Figure 2, the ZnO NPs agglomerates distributed in the sample are covered with a viscous layer. This aspect denotes the presence of the essential oil that “coated” the nanoparticles and, at the same time, encouraged the formation of agglomerates.

**Figure 8:**
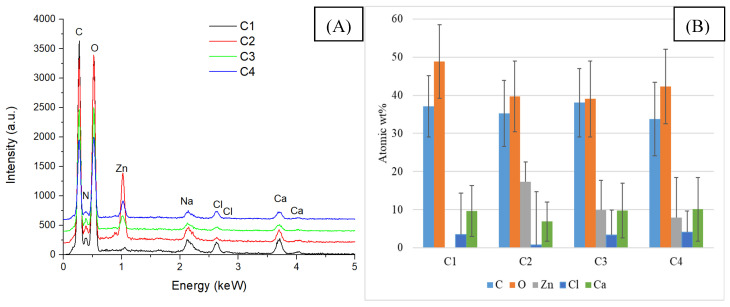
EDS analysis (**A**) and chemical composition (**B**) of composite samples included in group C.

In the case of the samples containing eucalyptus essential oil, no new elements are identified in the EDS analysis compared to the previously studied samples (A2, B2, C2), but the mass percentage of C and O increases because the essential oil is composed mainly of alcohols, terpenes, or acetates. The elemental composition of this group of samples is similar to that of the samples containing eucalyptus essential oil, the main difference being the presence of a higher amount of oxygen, as specified in Figure 8, due to the different composition of lavender essential oil.

**Figure 9:**
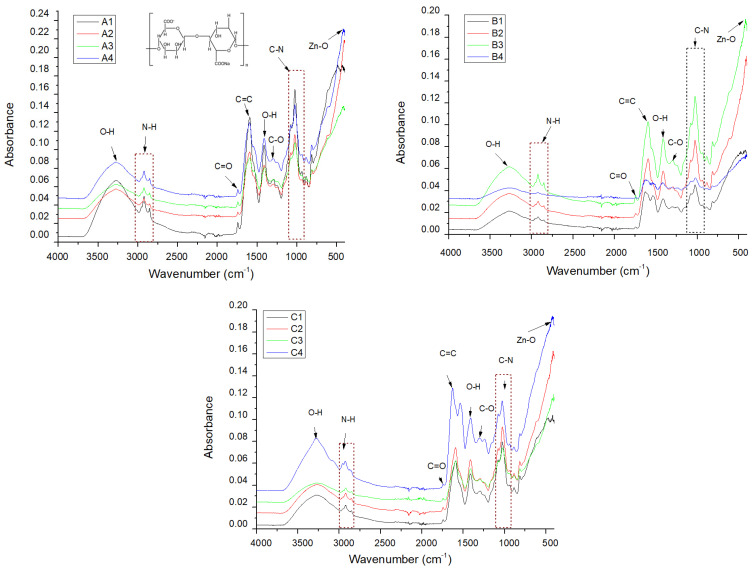
FTIR spectra for samples obtained with 2% (group A), 3% (group B), and 4% alginate (group C).

FTIR spectroscopy was used to identify the functional groups in the structure of the analyzed compounds; the absorption spectra are shown in Figure 9.

The FTIR spectra obtained for each group of samples show similar absorption maxima, the difference being due to the intensity of the maxima being directly proportional to the number of bonds present. The identification of the Zn–O bond confirms the presence of ZnO NPs (Figure 9, Table 1). The composition of egg white includes specific amino acid bonds present in its proteins, such as N-H, C=O, C=C, and C-N. The presence of the O–H bond may be due to the incorporation of alginate, for which the C–O bond is also specific, of essential oils, or due to accidental wetting of the samples. Additionally, the characteristic bands of the main constituents of essential oils overlap with the polymeric matrix, results that are linked to other studies [36,37,38].

The minor variations in the characteristic peaks of the identified bonds (summarized in Table 2) suggest that alginate and egg white do not chemically interact with each other. Instead, the observed behavior likely stems from alginate reacting with Ca^2^⁺ ions from calcium chloride to form calcium alginate.

**Figure 10:**
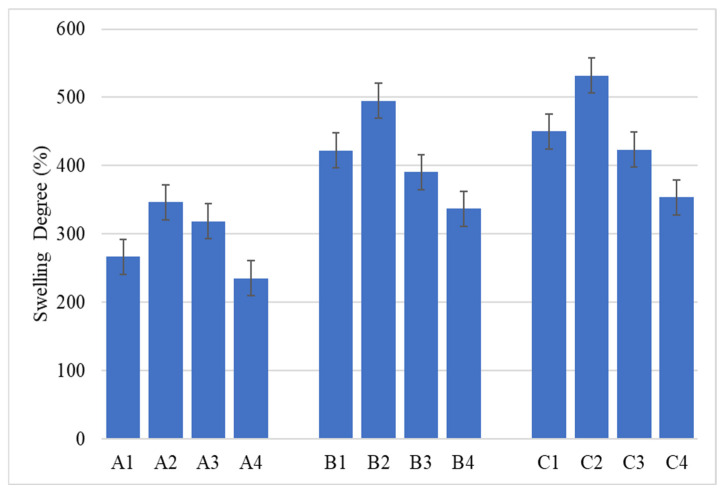
Swelling capacity for samples obtained with 2% (group A), 3% (group B), and 4% alginate (group C).

Since using a material that exhibits the ability to absorb water and water-soluble physiological fluids can keep the wound hydrated and promote faster healing, the swelling capacity of the synthesized composites was analyzed. The results were grouped in Figure 10, depending on the components (without ZnO, with ZnO, with ZnO coated with eucalyptus oil, and with ZnO coated with lavender oil), thus highlighting the effects of increasing alginate concentration on the swelling degree.

As can be seen in Figure 10, samples containing 2% alginate showed the lowest degree of swelling, while samples containing 4% alginate showed the highest degree of swelling. This behavior may be due to the fact that, with the introduction of a larger amount of alginate in the composite mass, after crosslinking and freeze-drying, the resulting structure is more porous, making it possible to capture a more significant amount of water [39,40]. This information is also confirmed by the SEM images obtained for samples that do not contain ZnO NPs (A1, B1, C1), indicating that composite C1 presents microporosity, which facilitates better liquid absorption.

**Figure 11:**
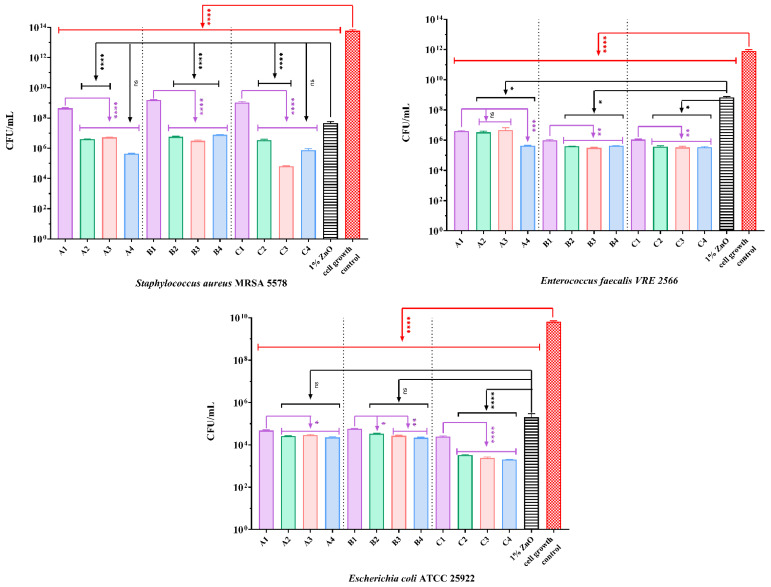
The anti-adherence capacity of composite materials against *S. aureus*, *E. faecalis*, and *E. coli*. The CFU/mL values were represented in logarithmic units. The differences between the samples tested and the cell growth control were statistically analyzed using one-way ANOVA and Dunnett’s multiple comparisons test. The comparisons between alginate/egg white membranes and alginate/egg white–ZnO were analyzed using one-way ANOVA and Holm–Šídák’s multiple comparisons test. The comparisons between ZnO and alginate/egg white–ZnO were analyzed following the same tests. The differences between alginate/egg white–ZnO membranes were not significant. (ns—not significant; * *p* < 0.05; ** *p* < 0.01; *** *p* < 0.001; **** *p* < 0.0001).

The antibacterial capacity of the alginate/egg white/ZnO membranes obtained in this study was performed by determining the colony-forming units/mL (CFU/mL) values. Antibiofilm activity was evaluated against two clinically isolated strains (methicillin-resistant *S. aureus* and vancomycin-resistant *Enterococcus*), and one standard strain (*E. coli* ATCC 25922), as these are among the most prevalent bacterial species in chronic wounds. Figure 11 shows the inhibitory effect, with a significant decrease (more than 10^5^ CFU/mL) for all samples compared to the cell growth control.

ZnO-loaded composites show moderately significant antibacterial effects compared to alginate/egg white membranes (A1, B1, and C1) but are higher than 1% ZnO suspension. Moreover, synergistic effects can be observed between ZnO NPs and the other bioactive compounds in the composite materials. A4 and C3 had the ability to inhibit the adhesion of *S. aureus* MRSA 5578 on their surfaces, as indicated by the lowest CFU/mL values.

The composites strongly influenced vancomycin-resistant *E. faecalis* development, decreasing the ability of Gram-positive bacteria to adhere to surfaces with more than 10^6^ CFU/mL. Moreover, comparing the resulting data, a synergistic effect between alginate and ZnO NPs can be observed for samples containing ZnO [41].

The materials induced the highest antibacterial activity against the *E. coli* strain. The significant susceptibility of *E. coli* under the action of the samples studied was higher than that of Gram-positive bacteria. Figure 11 shows significant differences between the groups. Also, as with *E. faecalis*, ZnO-containing composites showed a synergistic effect between ZnO and alginate. Groups A2, A3, A4, and B2 showed a moderate bacteriostatic impact against the tested strain, followed by B3 and B4. C2, C3, and C4 showed the most significant influence on the growth of Gram-negative bacteria. In addition, the samples loaded with ZnO NPs coated with essential oils exhibit a more significant decrease in CFU/mL than A2, B2, and C2. However, no notable differences were observed between the nanocomposites containing lavender or eucalyptus essential oil. In another study [35]–ZnO nanocomposite films presented significant antibacterial activity against *S. aureus* and *E. coli* and could potentially be used as a wound-healing material. Likewise, in their study, Trandafilovic et al. [42] demonstrated the great antibacterial activity of ZnO–alginate nanocomposite against the same strains. Likewise, in another study [39], the ZnO–alginate 3D-printed hydrogels showed a moderate inhibitory effect on *S. epidermidis*.

**Figure 12:**
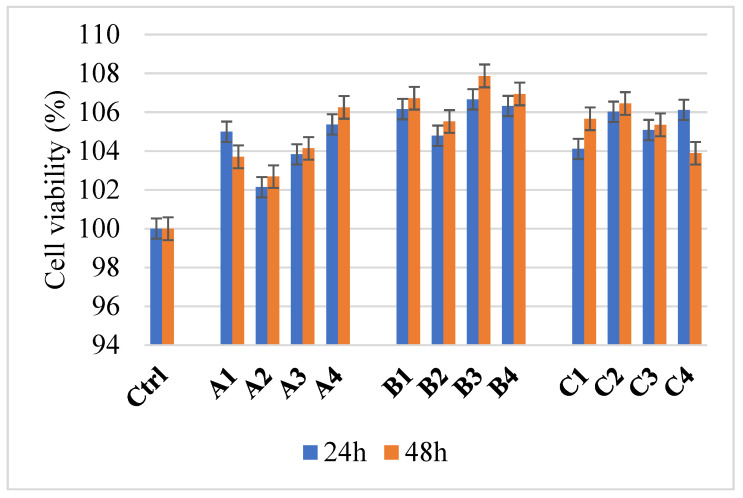
MTT assay for samples obtained with 2% (group A), 3% (group B), and 4% alginate (group C). Ctrl represents the cell’s control.

Considering the significant anti-adherence properties of developed nanocomposites, it is crucial for the synthesized dressing to enhance tissue healing, particularly cell proliferation. Accordingly, MTT cell viability tests for biocompatibility assessment of the materials in cell culture were performed using the HUVEC cell line, with the results displayed in Figure 12.

According to the results obtained, all four synthesized dressings exhibit cell viability higher than 100%, at both 24 and 48 h. The enhancement of cell proliferation and adhesion of human umbilical vein endothelial cells can be attributed, in particular, to the biological properties of albumin from egg white. Albumin serves as a substrate for cell attachment, is a valuable source of essential amino acids, helps to increase the growth rate, maintains osmotic balance, and stabilizes the pH. Furthermore, several studies suggest that albumin combined with sodium alginate enhances the bioavailability of alginate hydrogels [43,44,45,46].

In the case of sample A2, it was observed that the introduction of nanoparticles in the composition produced a decrease in viability. However, with the addition of essential oils, a maximum level of viability was reached. In group B, integrating a higher amount of alginate resulted in increased cell viability compared to group A samples. Again, the composite containing simple ZnO has a lower percentage of live cells, which is due to the possible cytotoxic effect of ZnO [47], but the use of essential oil potentiates the increase in cell viability [48]. The highest viability value was observed for sample B3 at 48 h. Samples containing 4% alginate show higher cell viability values than the control sample, as do the other groups (A, B). Excluding sample C4, the different composite materials show a slight increase in viability after 48 h compared to 24.

Considering the antimicrobial properties of nanocomposites and the biocompatibility results, the samples containing 4% alginate demonstrate significant potential for biomedical applications in wound healing. Further research will investigate the advanced biocompatibility of the developed materials, particularly through in vivo studies.

## 3. Conclusions

In this work, sodium alginate and egg white were utilized to synthesize a hydrogel, which was then combined with ZnO nanoparticles, with or without essential oil, and subsequently lyophilized to obtain a dressing that promotes burn healing. Each sample was characterized quantitatively and qualitatively using multiple analysis methods such as scanning electron microscopy, Fourier transform infrared spectroscopy, and X-ray dispersive spectroscopy. Furthermore, cell viability and the antibacterial properties of the hydrogels developed were also studied. Following the tests performed, it was observed that all the dressings obtained present porosity and a degree of swelling greater than 200% after three days. These characteristics are important in the design of an ideal dressing for treating burns, as the high porosity allows the exchange of oxygen and water between the wound and the outside, which ensures the maintenance of a moist environment for a longer period of time, which reduces the possibility of scar formation. Antibacterial assessments indicated that all samples reduced the ability of bacteria to adhere to composite surfaces, with the most significant results observed in group C, which contains 4% alginate in its composition. This is most likely due to the antimicrobial properties of alginate.

In conclusion, the obtained composites possess optimal characteristics for use as a dressing for treating burns, such as providing or maintaining a moist environment, allowing gas exchange between the injured tissue and the environment, providing protection against bacterial infections, not adhering to the wound, and being easy to remove.

## 4. Materials and Methods

### 4.1. Materials

Zinc nitrate hexahydrate (Zn(NO_3_)_2_ × 6H_2_O, 98%- reagent grade, CAS No. 10196-18-6), sucrose (C_12_H_22_O_11_, ≥99.5% purity, CAS No. 57-50-1), ethanol (≥99.9%, suitable for liquid chromatography, CAS No. 64-17-5), sodium alginate (medium viscosity, quality control: 200, CAS No. 9005-38-3), calcium chloride (CaCl_2_, ≥96.0% purity, CAS No. 10043-52-4), Nutrient Broth No. 2 (NB), agar (suitable for microbiology, CAS No. 9002-18-0), phosphate-buffer saline (PBS), isopropanol (≥99.9%, suitable for liquid chromatography, CAS No. 67-63-0), dimethyl sulphoxide (DMSO, for chromatography assays, CAS No. 67-68-5), Trypsin-EDTA, and [3-(4,5-dimethylazol-2-yl)-2,5-diphenyltetrazolium bromide] (MTT, CAS No. 298-93-1) were purchased from Sigma-Aldrich (Darmstadt, Germany). Lavender and eucalyptus essential oils were acquired from Roth (Karlsruhe, Germany), fetal bovine serum from EuroClone (Pero, Italy), and penicillin–streptomycin solution from PAN Biotech (Aidenbach, Germany). All chemicals were used without further purification. The egg whites used in the development of the composites come from eggs purchased from Lidl, Romania. The strains tested in this study are provided from the Microorganisms Collection of the Department of Microbiology, Faculty of Biology and Research Institute of the University of Bucharest.

### 4.2. Experimental Procedure

#### 4.2.1. Synthesis of ZnO Nanoparticles

The synthesis method chosen to obtain ZnO NPs is the combustion method previously described by Md. Tariqul Islam et al. [49], with sucrose as a fuel reagent.

**Figure 13:**
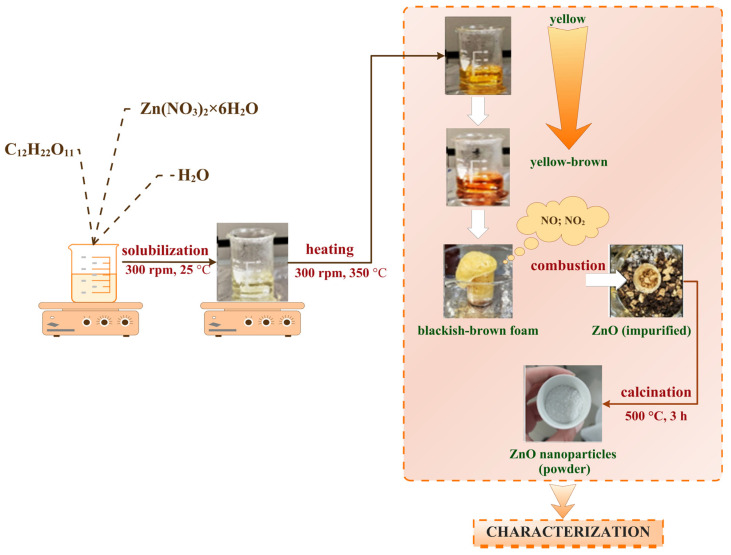
Technological flow for obtaining zinc oxide nanoparticles. Designed with ConceptDraw Diagram 16.

ZnO NPs were obtained by making a solution containing 10.709 g Zn(NO_3_)_2_ × 6 H_2_O, 3.6 g sucrose, and 9 mL distilled water. The obtained mixture was heated on a hot plate at 300–350 °C under continuous stirring. The decomposition of zinc nitrate produced yellow-brown gases, a result of the release of nitrogen oxides (NO, NO_2_), eventually leading to the formation of a blackish-brown foam. The foam was stirred for approximately 20 min, allowing the carbon present to combust and form carbon dioxide, which yielded a yellowish white ZnO powder. The next step involved placing the powder and any unreacted foam into a furnace at 500 °C for 3 h. After the thermal treatment (calcination), a white powder was obtained and subsequently characterized. The complete technological flow of the synthesis process is illustrated in Figure 13.

Two essential oils were used to enhance the synergistic effect of ZnO particles. Thus, lavender and eucalyptus essential oils were selected from the literature and were deposited on the previously obtained nanoparticles. For this, 0.5 mL of essential oil was added to 10 mL of ethanol and poured, under stirring, over a suspension consisting of 0.5 g of ZnO and 0.5 mL of ethanol. After homogenization, the suspension was dried for 24 h to evaporate the ethanol. Thus, in addition to the ZnO sample, two more powders were obtained, known as ZnO_L_ and ZnO_E_, where L represents lavender oil and E represents eucalyptus oil.

#### 4.2.2. Development of Alginate/Egg White–Zinc Oxide Composite

In order to develop composite materials, a mixture of alginate and commercial egg white was used. To analyze the influence of the amount of alginate incorporated in the composite, samples with different percentages of alginate dissolved in 100 mL of commercial egg white were obtained. Thus, 3 classes of composite materials were obtained, divided as follows: samples from group A containing 2 wt.% alginate, samples from group B with 3 wt.% alginate, and samples from group C with 4 wt.% alginate. Four samples were performed for each group. The first sample contains only alginate and egg white in the percentages specific to each group; for the second, third, and fourth samples, respectively, ZnO NPs, ZnO coated with lavender essential oil, and ZnO coated with eucalyptus essential oil were introduced in a proportion of 0.5 wt.% of the homogeneous solution formed from egg white and alginate. The essential oils were incorporated/added to enhance the biological activity of the composites.

The composition and notation used for the obtained samples are summarized in Table 2.

The crosslinking process for obtaining dressing composites depends on the ratio of CaCl_2_ to alginate. The concentration of the crosslinking solution was adjusted for each group according to the guideline: 0.5 moles of CaCl_2_ per total moles of alginate.

First, the specific amount of calcium chloride was solubilized in distilled water, and, under continuous ultrasonication, the amount of ZnO NPs was added. This suspension was added over a solution obtained from sodium alginate and egg white, with a concentration of 2, 3, or 4%, specific to each sample group, as listed in Table 1. The hydrogel thus obtained was homogenized under continuous stirring for 30 min. A 0.5 M calcium chloride solution was added to the gels to achieve complete crosslinking. The resulting hydrogel was poured into Petri dishes and washed several times to remove the excess CaCl_2_. Finally, the samples were subjected to an accelerated cooling treatment at −15 °C. The freeze-drying process was conducted using a LABCONCO lyophilizer—FreeZone 2.5 Liter Freeze Dry Systems (Kansas City, MO, USA) to preserve the bioactive compounds, improve the mechanical properties, increase the porosity of the dressing, and perform further tests on it. This process included pre-freezing, followed by freeze-drying at −85 °C. Initially, the primary freezing phase occurred at −40 °C for 3 h, followed by vacuum freezing at approximately 0.13 mBar for 4 h. Subsequently, the drying process continued at a higher temperature and pressure (up to −85 °C, 0.002 mBar) for 24 h.

### 4.3. Characterization Methods

The crystallinity of the obtained material was investigated by X-ray diffraction using a PANalytical Empyrean diffractometer, equipped with a hybrid monochromator (2xGe 220) on the incident side and a parallel plate collimator mounted on a PIXcel 3D detector on the diffracted side. The XRD measurements with a grazing incidence angle were performed at room temperature, with an incidence angle of ω = 0.5° for Bragg angle values of 2θ between 10° and 80°, with a step of 0.025256 and an acquisition time of 255 s, using Cu Kα radiation with λ = 1.5406 Å (40 mA and 45 kV). The average crystallite size (D) was determined using Scherrer’s Equation (1).

(1) $$D=\frac{k\lambda }{\beta \;\cos\theta }$$

where β is full width at half maximum (FWHM); θ is Bragg’s angle; λ is wavelength of the X-ray; and D is the crystallite size.

To investigate the functional group characteristic of the synthesized materials, a small amount of the synthesized composites was analyzed using a Thermo iN10-MX Fourier transform (FT)-IR microscope (Thermo Fischer Scientific, Waltham, MA, USA). Measurements were performed at room temperature, with 64 scans of the sample between 4000 and 400 cm^−1^, at a resolution of 4 cm^−1^. The recording of the information thus acquired was possible by connecting the spectrometer to a data acquisition and processing unit, using the Omnic software (version 8.2 Thermo Nicolet).

SEM-EDS characterization was performed using a QUANTA INSPECT F50 scanning electron microscope purchased from FEI (Hillsboro, OR, USA), equipped with a field emission electron gun (FEG) with a resolution of 1.2 nm and an energy dispersive X-ray spectrometer (EDS) with a resolution at MnK of 133 eV. All samples were analyzed under the same conditions. In order to investigate the morphology and size of the materials obtained, the samples were fixed on a slide using carbon tape and introduced under vacuum into the analysis chamber of a scanning electron microscope. The images obtained were made by recording the resulting secondary electron beam, as well as backscattered electrons, with an energy of 30 keV. The size of the NPs was measured using ImageJ software (version 1.8.0, National Institutes of Health, Madison, WI, USA).

The swelling degree in simulated body fluid (SBF) was determined at room temperature as the ratio between the amount of liquid contained in the hydrogel after the swelling treatment and the mass of the dry polymer (xerogel), according to Formula (2):


(2) $$\mathrm{Swelling}\;\mathrm{degree}=[\frac{\left(mo-mt\right)}{mt}]\times 100$$

In this assay, samples from each freeze-dried composite were initially weighed dry (*mo*), followed by an evaluation of their mass after being immersed in SBF at room temperature for 3 days (*mt*). The SBF was prepared according to the recipe proposed by Kokubo et al. [50].

### 4.4. Microbiological Activity

The anti-adherent capacity of the composite obtained in this study was performed by determining the colony-forming units/mL values (CFU/mL). The antibacterial activity was quantitatively evaluated against the *Staphylococcus aureus* MRSA 5578 (methicillin-resistant *S. aureus*, clinical isolate), *Enterococcus faecalis* VRE 2566 (vancomycin-resistant *Enterococcus*, clinical isolate), and *Escherichia coli* ATCC 25922. The impact of the contaminants on the experiment was mitigated by previously sterilizing all samples (0.5 cm × 0.5 cm) by maintaining UV radiation exposure for 30 min on each side. The sterility test was performed for each sample by maintaining it in the nutrient broth media for 24 h at 37 °C to confirm the sterility of the tested samples before the antimicrobial assay. The clearness of the broth media confirmed the sterility of the samples. Furthermore, UV radiation activates the ZnO NPs from the structure of the membranes [51,52,53].

Bacterial cell suspensions (1.5 × 10^8^ CFU/mL) were performed in a sterile physiological buffer saline from fresh cultures (20–24 h). The quantitative evaluation of the antibacterial activity of each sample was performed using a nutrient broth: bacterial cell suspension ratio = 10:1, and the final density of 1.5 × 10^7^ CFU/mL. The anti-adherent capacity/biofilm development of the composite membranes was performed using the method described in the previous studies [54,55] and according to the CLSI standard [56]. The negative control was performed, and it considered sterile media. The positive control consisted of broth media inoculated with bacterial suspensions. Also, the alginate–egg white membranes and a suspension with 1% ZnO were performed as controls, following the same method. The viable colony formation was expressed as CFU (colony-forming units)/mL. The CFU/mL values were expressed as the mean of the total number of colonies × 1/D (D = decimal dilution, for which the number of total colonies was determined) [54,55]. The assays were performed in three independent experiments.

### 4.5. Cell Viability Determination

The human umbilical vein endothelial cells (HUVEC) cell line was used to evaluate cell viability under the action of the developed composite materials. The cell culture was maintained at 37 °C in a humidified atmosphere with 5% CO_2_, and the growth medium used was MEM, supplemented with 10% fetal bovine serum. An inverted phase-contrast microscope was used to visualize the cells and monitor the different growth stages. To culture the cells, which were maintained at −80 °C in 95% fetal bovine serum and 5% DMSO, they were thawed in a water bath at 37 °C. They were trypsinized and transferred to tubes containing fresh pre-warmed medium. The cell pellet for seeding fibroblasts is obtained by centrifuging both frozen and adherent cells for 10 min at 1500 rpm at 180 °C. Subsequently, the supernatant is discarded, the cell pellet is resuspended in the growth medium, and the cells are counted using a Burker–Turk counting chamber. Samples were sterilized by exposure to an ultraviolet lamp for 1 h.

The MTT assay is based on the study of dehydrogenase activity. The principle of this method involves reducing the yellow-colored compound MTT to a dark blue formazan. The reduction coefficient is directly proportional to the number of viable cells, representing the cellular or mitochondrial integrity index. After removing the medium, the cells were washed with PBS. MTT solution was added, followed by incubation at 37 °C for two hours in the dark. The MTT solution was removed, and after adding the same volume of isopropanol, the formazan crystals were resuspended by pipetting until they were completely dissolved. The absorbance was measured spectrophotometrically at a wavelength of 595 nm using a SpectraMax i3x Multi-Mode microplate reader provided by Molecular Devices (San Jose, CA, USA) [57].

### 4.6. Statistical Analysis

The data results were statistically analyzed using GraphPad Prism, version 10.4, from GraphPad Software (San Diego, CA, USA). All experiments were performed in three independent determinations. The results are expressed as ±SD (standard deviation) and analyzed using a one-way analysis of variance (one-way ANOVA) followed by a multiple comparisons assay according to the experimental method. The differences between groups/samples were considered statistically significant when the *p*-value was <0.05.

## Figures and Tables

**Table 1:** Assignment of relevant IR adsorption bands of alginate-based composites.

Sample/Assignment	A1/B1/C1	A2/B2/C2	A3/B3/C3	A4/B4/C4
O–H bond	3272/3277/3270	3270/3270/3271	3269/3270/3268	3273/3272/3279
N–H stretch	2924/2923/2925	2924/2922/2923	2924/2923/2925	2923/2923/2926
C=O stretch	1745/1743/1745	1744/1744/1745	1745/1745/1744	1744/1745/1746
C=C	1595/1597/1593	1595/1593/1593	1593/1591/1592	1596/1595/1630
O–H	1413/1412/1412	1412/1411/1411	1410/1412/1412	1413/1409/1409
C–N	1027/1025/1025	1027/1026/1026	1026/1025/1026	1028/1024/1027
Zn–O	/-/-	416/417/413	417/418/426	417/421/421

**Table 2:** Name and composition of the composite materials obtained.

Composite Name	Egg White (mL)	Alginate (%)	ZnO (%)	Essential Oil Type
A1	100	2	0	-
A2			0.5	-
A3			0.5	Lavender
A4			0.5	Eucalyptus
B1	100	3	0	-
B2			0.5	-
B3			0.5	Lavender
B4			0.5	Eucalyptus
C1	100	4	0	-
C2			0.5	-
C3			0.5	Lavender
C4			0.5	Eucalyptus

## Data Availability

The original contributions presented in this study are included in the article. Further inquiries can be directed to the corresponding author.

## References

[1] Bueno J., Demirci F., Baser K.H.C. (2017). Antimicrobial Strategies in Novel Drug Delivery Systems. The Microbiology of Skin, Soft Tissue, Bone and Joint Infections.

[2] Linz M.S., Mattappallil A., Finkel D., Parker D. (2023). Clinical Impact of Staphylococcus aureus Skin and Soft Tissue Infections. Antibiotics.

[3] Mihai M.M., Preda M., Lungu I., Gestal M.C., Popa M.I., Holban A.M. (2018). Nanocoatings for Chronic Wound Repair—Modulation of Microbial Colonization and Biofilm Formation. Int. J. Mol. Sci..

[4] Kaushik M., Niranjan R., Thangam R., Madhan B., Pandiyarasan V., Ramachandran C., Oh D.-H., Venkatasubbu G.D. (2019). Investigations on the antimicrobial activity and wound healing potential of ZnO nanoparticles. Appl. Surf. Sci..

[5] Bessa L.J., Fazii P., Di Giulio M., Cellini L. (2015). Bacterial isolates from infected wounds and their antibiotic susceptibility pattern: Some remarks about wound infection. Int. Wound J..

[6] Khorasani M.T., Joorabloo A., Adeli H., Milan P.B., Amoupour M. (2021). Enhanced antimicrobial and full-thickness wound healing efficiency of hydrogels loaded with heparinized ZnO nanoparticles: In vitro and in vivo evaluation. Int. J. Biol. Macromol..

[7] U.S. Food and Drug Administration (2024). Title 21—Food and Drugs, Chapter I—Food and Drug Administration Department of Health and Human Services, Subchapter B—Food for Human Consumption, Part 182 Substances Generally Recognized as Safe.

[8] Spoială A., Ilie C.-I., Trușcă R.-D., Oprea O.-C., Surdu V.-A., Vasile B.Ș., Ficai A., Ficai D., Andronescu E., Dițu L.-M. (2021). Zinc Oxide Nanoparticles for Water Purification. Materials.

[9] Jin S.-E., Jin H.-E. (2021). Antimicrobial Activity of Zinc Oxide Nano/Microparticles and Their Combinations against Pathogenic Microorganisms for Biomedical Applications: From Physicochemical Characteristics to Pharmacological Aspects. Nanomaterials.

[10] Gudkov S.V., Burmistrov D.E., Serov D.A., Rebezov M.B., Semenova A.A., Lisitsyn A.B. (2021). A Mini Review of Antibacterial Properties of ZnO Nanoparticles. Front. Phys..

[11] Siddiqi K.S., Rahman A.U., Tajuddin, Husen A. (2018). Properties of Zinc Oxide Nanoparticles and Their Activity Against Microbes. Nanoscale Res. Lett..

[12] Stoica A.E., Bîrcă A.C., Pițigoi M.L., Grumezescu A.M., Vasile B.Ș., Iordache F., Ficai A., Andronescu E. (2024). Nanostructured Zinc Oxide for Wound Dressings. UPB Sci. Bull. Ser. B Chem. Mater. Sci..

[13] Zudyte B., Luksiene Z. (2021). Visible light-activated ZnO nanoparticles for microbial control of wheat crop. J. Photochem. Photobiol. B.

[14] Gao Y., Han Y., Cui M., Tey H.L., Wang L., Xu C. (2017). ZnO nanoparticles as an antimicrobial tissue adhesive for skin wound closure. J. Mater. Chem. B.

[15] Rayyif S.M.I., Mohammed H.B., Curuțiu C., Bîrcă A.C., Grumezescu A.M., Vasile B.Ș., Dițu L.M., Lazăr V., Chifiriuc M.C., Mihăescu G. (2021). ZnO Nanoparticles-Modified Dressings to Inhibit Wound Pathogens. Materials.

[16] Jiang J., Pi J., Cai J. (2018). The Advancing of Zinc Oxide Nanoparticles for Biomedical Applications. Bioinorg. Chem. Appl..

[17] Espitia P.J.P., Otoni C.G., Soares N.F.F. (2016). Zinc Oxide Nanoparticles for Food Packaging Applications. Antimicrobial Food Packaging.

[18] Pushpalatha C., Suresh J., Gayathri V., Sowmya S., Augustine D., Alamoudi A., Zidane B., Albar N.H.M., Patil S. (2022). Zinc Oxide Nanoparticles: A Review on Its Applications in Dentistry. Front. Bioeng. Biotechnol..

[19] Hatamie A., Khan A., Golabi M., Turner A.P.F., Beni V., Mak W.C., Sadollahkhani A., Alnoor H., Zargar B., Bano S. (2015). Zinc Oxide Nanostructure-Modified Textile and Its Application to Biosensing, Photocatalysis, and as Antibacterial Material. Langmuir.

[20] Mendes C.R., Dilarri G., Forsan C.F., de Moraes Ruy Sapata V., Lopes P.R.M., de Moraes P.B., Montagnolli R.N., Ferreira H., Bidoia E.D. (2022). Antibacterial action and target mechanisms of zinc oxide nanoparticles against bacterial pathogens. Sci. Rep..

[21] Álvarez-Chimal R., García-Pérez V.I., Álvarez-Pérez M.A., Tavera-Hernández R., Reyes-Carmona L., Martínez-Hernández M., Arenas-Alatorre J.Á. (2022). Influence of the particle size on the antibacterial activity of green synthesized zinc oxide nanoparticles using Dysphania ambrosioides extract, supported by molecular docking analysis. Arab. J. Chem..

[22] Asadpoor M., Ithakisiou G.-N., van Putten J.P.M., Pieters R.J., Folkerts G., Braber S. (2021). Antimicrobial Activities of Alginate and Chitosan Oligosaccharides Against Staphylococcus aureus and Group B Streptococcus. Front. Microbiol..

[23] Sayin S., Depci T., Naz M., Sezer S., Karaaslan M.G. (2022). Characterization and evaluation of the antimicrobial properties of algal alginate; a potential natural protective for cosmetics. J. Res. Pharm..

[24] Zhang M., Zhao X. (2020). Alginate hydrogel dressings for advanced wound management. Int. J. Biol. Macromol..

[25] Jalili-Firoozinezhad S., Rajabi-Zeleti S., Mohammadi P., Gaudiello E., Bonakdar S., Solati-Hashjin M., Marsano A., Aghdami N., Scherberich A., Baharvand H. (2015). Facile Fabrication of Egg White Macroporous Sponges for Tissue Regeneration. Adv. Healthc. Mater..

[26] Aiyelabegan H.T., Zaidi S.S.Z., Fanuel S., Eatemadi A., Ebadi M.T.K., Sadroddiny E. (2016). Albumin-based biomaterial for lung tissue engineering applications. Int. J. Polym. Mater. Polym. Biomater..

[27] Jalili-Firoozinezhad S., Filippi M., Mohabatpour F., Letourneur D., Scherberich A. (2020). Chicken egg white: Hatching of a new old biomaterial. Mater. Today.

[28] Jahani S., Ashrafizadeh H., Babai K., Siahpoosh A., Cheraghian B. (2019). Effect of ointment-based egg white on healing of second- degree wound in burn patients: A triple-blind randomized clinical trial study. Avicenna J. Phytomed.

[29] Deyno S., Mtewa A.G., Abebe A., Hymete A., Makonnen E., Bazira J., Alele P.E. (2019). Essential oils as topical anti-infective agents: A systematic review and meta-analysis. Complement. Ther. Med..

[30] Khajavi R., Abbasipour M., Barzi M.G., Rashidi A., Rahimi M.K., Mirzababa H.H. (2014). Eucalyptus Essential Oil-Doped Alginate Fibers as a Potent Antibacterial Wound Dressing. Adv. Polym. Technol..

[31] Travassos A.R., Claes L., Boey L., Drieghe J., Goossens A. (2011). Non-fragrance allergens in specific cosmetic products. Contact Dermat..

[32] de Groot A.C., Schmidt E. (2021). Chemical Composition of and Contact Allergy to Essential Oils. Essential Oils.

[33] Mori H.-M., Kawanami H., Kawahata H., Aoki M. (2016). Wound healing potential of lavender oil by acceleration of granulation and wound contraction through induction of TGF-β in a rat model. BMC Complement. Altern. Med..

[34] Huang J., You X., Xin P., Gu Z., Chen C., Wu J. (2021). Egg white as a natural and safe biomaterial for enhanced cancer therapy. Chin. Chem. Lett..

[35] M A.M., D U., Ashwin B.M., Yardily A., Dennison M.S. (2025). Microwave-assisted green synthesized ZnO nanoparticles: An experimental and computational investigation. Discov. Appl. Sci..

[36] Wang H., Ren C., Bai W., Tang Z., Lei H., Tian H., Du G. (2025). Eucalyptus oil encapsulated within calcium-crosslinked sodium alginate for natural wood preservatives against fungi and termite. Ind. Crops Prod..

[37] Rusu A.G., Niță L.E., Roșca I., Croitoriu A., Ghilan A., Mititelu-Tarțău L., Grigoraș A.V., Crețu B.E.B., Chiriac A.P. (2023). Alginate-Based Hydrogels Enriched with Lavender Essential Oil: Evaluation of Physicochemical Properties, Antimicrobial Activity, and In Vivo Biocompatibility. Pharmaceutics.

[38] Sánchez E.C., García M.T., Pereira J., Oliveira F., Craveiro R., Paiva A., Gracia I., García-Vargas J.M., Duarte A.R.C. (2023). Alginate–Chitosan Membranes for the Encapsulation of Lavender Essential Oil and Development of Biomedical Applications Related to Wound Healing. Molecules.

[39] Cleetus C.M., Primo F.A., Fregoso G., Raveendran N.L., Noveron J.C., Spencer C.T., Ramana C.V., Joddar B. (2020). Alginate Hydrogels with Embedded ZnO Nanoparticles for Wound Healing Therapy. Int. J. Nanomed. Vol..

[40] Li L., Chen Y., Wang Y., Shi F., Nie Y., Liu T., Song K. (2019). Effects of concentration variation on the physical properties of alginate-based substrates and cell behavior in culture. Int. J. Biol. Macromol..

[41] Jayakumar R., Kumar P.S., Mohandas A., Lakshmanan V.-K., Biswas R. (2015). Exploration of alginate hydrogel/nano zinc oxide composite bandages for infected wounds. Int. J. Nanomed..

[42] Trandafilović L.V., Božanić D.K., Dimitrijević-Branković S., Luyt A.S., Djoković V. (2012). Fabrication and antibacterial properties of ZnO–alginate nanocomposites. Carbohydr. Polym..

[43] Francis G.L. (2010). Albumin and mammalian cell culture: Implications for biotechnology applications. Cytotechnology.

[44] Cascone M.G., Rosellini E., Maltinti S., Baldassare A., Lazzeri L. (2018). Cell laden alginate/albumin hydrogel fibers for potential skin tissue engineering applications. Biomed. Eng..

[45] Yuan H., Zheng X., Liu W., Zhang H., Shao J., Yao J., Mao C., Hui J., Fan D. (2020). A novel bovine serum albumin and sodium alginate hydrogel scaffold doped with hydroxyapatite nanowires for cartilage defects repair. Colloids Surf. B Biointerfaces.

[46] Meng R., Zhu H., Deng P., Li M., Ji Q., He H., Jin L., Wang B. (2023). Research progress on albumin-based hydrogels: Properties, preparation methods, types and its application for antitumor-drug delivery and tissue engineering. Front. Bioeng. Biotechnol..

[47] Mendes L.P., Delgado J.M.F., Costa A.D.A., Vieira M.S., Benfica P.L., Lima E.M., Valadares M.C. (2015). Biodegradable nanoparticles designed for drug delivery: The number of nanoparticles impacts on cytotoxicity. Toxicol. Vitr..

[48] Miastkowska M., Kantyka T., Bielecka E., Kałucka U., Kamińska M., Kucharska M., Kilanowicz A., Cudzik D., Cudzik K. (2021). Enhanced biological activity of a novel preparation of lavandula angustifolia essential oil. Molecules.

[49] Islam M.T., Dominguez A., Alvarado-Tenorio B., Bernal R.A., Montes M.O., Noveron J.C. (2019). Sucrose-Mediated Fast Synthesis of Zinc Oxide Nanoparticles for the Photocatalytic Degradation of Organic Pollutants in Water. ACS Omega.

[50] Kokubo T., Kushitani H., Sakka S., Kitsugi T., Yamamuro T. (1990). Solutions able to reproduce in vivo surface-structure changes inbioactive glass-ceramic A-W. J. Biomed. Mater. Res..

[51] Asmat-Campos D., Rojas-Jaimes J., de la Cruz M.S., de Oca-Vasquez G.M. (2024). Enhanced antimicrobial efficacy of biogenic ZnO nanoparticles through UV-B activation: A novel approach for textile garment. Heliyon.

[52] Haddada E.B., Karkouch I., Hamraoui K., Faris N., Tabbene O., Horchani K., Ferhi M. (2023). Highly efficient antibacterial activity in the dark and under UV illumination of ZnO nanoplates dispersed in water. Emergent Mater..

[53] Jin S.E., Hwang W., Lee H.J., Jin H.-E. (2017). Dual UV irradiation-based metal oxide nanoparticles for enhanced antimicrobial activity in *Escherichia coli* and M13 bacteriophage. Int. J. Nanomed..

[54] Dolete G., Ilie C.-I., Chircov C., Purcăreanu B., Motelica L., Moroșan A., Oprea O.C., Ficai D., Andronescu E., Dițu L.-M. (2023). Synergistic Antimicrobial Activity of Magnetite and Vancomycin-Loaded Mesoporous Silica Embedded in Alginate Films. Gels.

[55] Lemnaru G.-M., Truşcă R.D., Ilie C.-I., Țiplea R.E., Ficai D., Oprea O., Stoica-Guzun A., Ficai A., Dițu L.-M. (2020). Antibacterial Activity of Bacterial Cellulose Loaded with Bacitracin and Amoxicillin: In Vitro Studies. Molecules.

[56] CLSI (2023). Performance Standards for Antimicrobial Susceptibility Testing, CLSI Supplement M100.

[57] Chircov C., Bîrcă A.C., Dănciulescu L.A., Neacșu I.A., Oprea O.C., Trușcă R.D., Andronescu E. (2023). Usnic Acid-Loaded Magnetite Nanoparticles—A Comparative Study between Synthesis Methods. Molecules.

